# A retropharyngeal–mediastinal hematoma with supraglottic and tracheal obstruction: The role of multidisciplinary airway management

**DOI:** 10.4103/0974-2700.70776

**Published:** 2010

**Authors:** Torsten Birkholz, Stefanie Kröber, Christian Knorr, Albert Schiele, Klaus Bumm, Joachim Schmidt

**Affiliations:** Department of Anesthesiology, University Hospital Erlangen, Krankenhausstr. 12, D-91054, Erlangen, Germany; 1 Department of Surgery, University Hospital Erlangen, Krankenhausstr. 12, D-91054, Erlangen, Germany; 2 Department of Otorhinolaryngology, University Hospital Erlangen, Krankenhausstr. 12, D-91054, Erlangen, Germany

**Keywords:** Airway obstruction, endotracheal, emergency medicine, intubation, tracheostomy, trauma center

## Abstract

A 77-year-old man suffered hypoxemic cardiac arrest by supraglottic and tracheal airway obstruction in the emergency department. A previously unknown cervical fracture had caused a traumatic retropharyngeal–mediastinal hematoma. A lifesaving surgical emergency tracheostomy succeeded. Supraglottic and tracheal obstruction by a retropharyngeal–mediastinal hematoma with successful resuscitation via emergency tracheostomy after hypoxemic cardiac arrest has never been reported in a context of trauma. This clinically demanding case outlines the need for multidisciplinary airway management systems with continuous training and well-implemented guidelines. Only multidisciplinary staff preparedness and readily available equipments for the unanticipated difficult airway solved the catastrophic clinical situation.

## INTRODUCTION

In a trauma patient, the unanticipated difficult airway with airway obstruction is connected to various pathologies and might be an immediate life-threatening situation. Among many possible causes of this rare condition, the presence of the retropharyngeal hematoma is always to be considered.[1–3] In this study, we describe the unique case of multidisciplinary airway management in a man with hypoxemic cardiac arrest due to supraglottic and tracheal obstruction, who had a previously unknown C2-fracture with an extensive retropharyngeal–mediastinal hematoma.

## CASE REPORT

In the late evening, the in-hospital resuscitation team received a cardiac arrest call from the emergency room. They found a 77-year-old man who already had been resuscitated by basic life support measures, and who currently had return of spontaneous circulation. He was admitted to the surgical emergency room with a leg fracture after a high velocity car crash about 4 h ago. During the conservative surgical treatment of the fracture, the patient suffered sudden severe dyspnea and subsequent hypoxic cardiac arrest. There were no other injuries known at that time, and he had showed no neurological impairment. The Anesthesiologist in training continued the manual in-line stabilization established during the resuscitation and immediately attempted intubation with a size three Macintosh blade and an 8 mm I.D. Magill endotracheal tube armed with a bougie, but could not identify any known laryngeal structure. Through a laryngeal mask, minimal ventilation and at least life-sustaining oxygenation was possible, with a SpO_2_ of about 85%. Immediately, two anesthesiology specialists arrived for the management of this unanticipated difficult airway. Due to the traumatic history of the patient, the surgeon-in-charge was also present.

**Figure 1:**
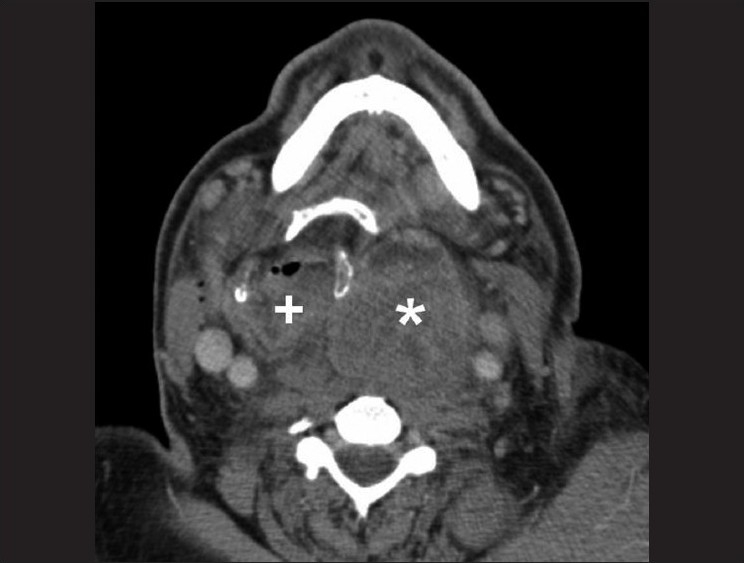
Retropharyngeal hematoma (*) grossly dislocating the larynx (+) to the right at the level of the hyoid

During ongoing laryngeal mask ventilation, the resuscitation team prepared for another intubation attempt. Laryngoscopy with a size four Macintosh blade revealed a grossly altered pharyngeal anatomy with circumferential hypopharyngeal swelling. The nodular altered and bleeding mucosa was reminiscent of a hypopharyngeal tumor. Laryngeal structures could not be identified. The outer neck showed a left-sided swelling and a marked dislocation of the larynx to the right [Figure 1], and especially the cricothyroideus membrane could not be identified clearly. The trauma surgeon resident present was instructed to prepare for emergency tracheostomy, as cricothyroidotomy was not considered possible.

After intermitting laryngeal mask ventilation, a fiberoptic intubation attempt with a flexible endoscope was not successful, and again, no laryngeal structures could be identified. Subsequently, no further ventilation was possible, and oxygen saturation was falling rapidly. The team leader requested emergency tracheostomy.

**Figure 2:**
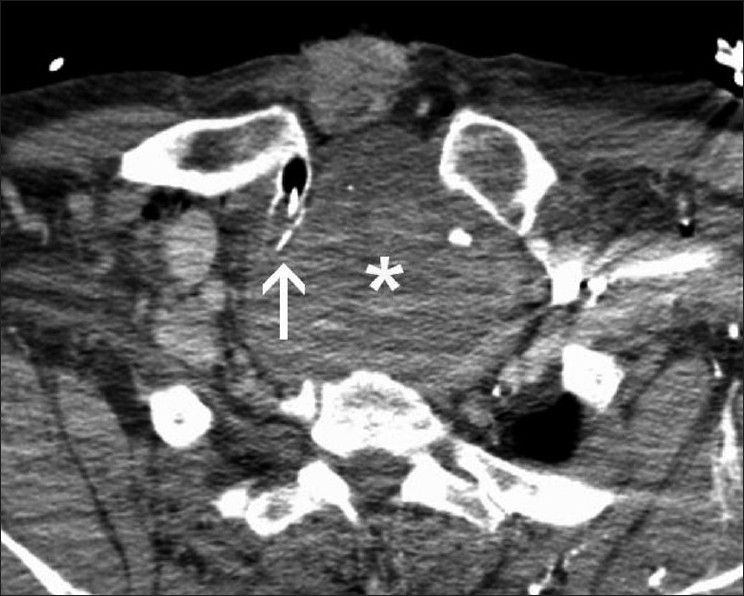
Endotracheal tube entering the tracheal lumen through tracheostomy (*arrow*), compressed by a large retropharyngeal–mediastinal hematoma (*) at the level of Th 2

In the meantime, an experienced surgeon had arrived and started the emergency tracheostomy. Tracheostomy was technically difficult, and hypoxemic cardiac arrest occurred. After a few minutes with intermitting thorax compression and 1 mg of adrenaline, tracheostomy of a grossly rightly dislocated and proximally compressed trachea succeeded with the insertion of a 6-mm I.D. Magill endotracheal tube. The site of tracheostomy was atypical and ranged into the upper thoracic aperture [Figure 2]. Ventilation was only possible with a deeply introduced endotracheal tube.

**Figure 3:**
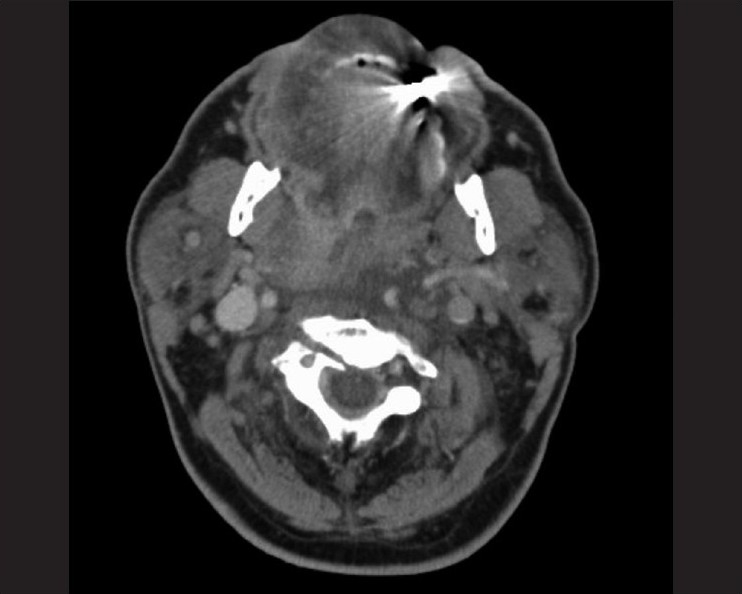
Transversal CT-scan of the C2-fracture

After reoxygenation, immediate return of spontaneous circulation was observed. In-line stabilization was continued by means of a collar. The subsequently performed CT scan revealed a fracture of C2 [Figure 3] with an extended retropharyngeal and mediastinal hematoma. The pharynx, the larynx, and the trachea were obstructed by the hematoma [Figures 1 and 2]. In the operating room, the emergency tracheostomy was converted into a permanent tracheostomy, and the patient received a halo fixator, which remained in place in the clinical course and during the following months. Due to the extensive tissue hemorrhage and the ongoing need for invasive positive pressure ventilation, surgical hematoma decompression was not considered and left to resorption. Unfortunately, the patient showed the clinical picture of partial tetraparesis. Assessment of the central nervous system by CT scan was severely limited by halo fixator artifacts, but it was suggestive for partial bilateral basal ganglia infarction. Therefore, partial tetraparesis was mainly considered a result of hypoxia, although a role of the initial trauma or the repeated intubation attempts could not be excluded. After about 1 month of intensive care therapy, the patient was transferred to a rehabilitation facility and was finally discharged home awake.

## DISCUSSION

Upper airway obstruction due to a retropharyngeal hematoma is a rare, but well-known entity. Airway obstruction due to a retropharyngeal hematoma mostly affects the upper airway. An analogous extent of obstruction has been reported before in a patient with acquired hemophilia A, where conventional intubation was possible.[1] To the best of our knowledge, this is the first case reported with both supraglottic and tracheal obstruction due to a traumatic retropharyngeal hematoma, which was successfully resuscitated by emergency tracheostomy. Traumatic retropharyngeal hematomas are generally rare, and could occur after trauma of any severity to the neck.[2,3] Hyperextension alone is an ample trauma in elder individuals.[3] Cervical fractures above the level of C6 are prone to develop a retropharyngeal hematoma,[4] so our patient was especially at risk. However, the clinical manifestation could be early with sudden airway obstruction during the first assessment or might be delayed for hours.[5–7] As displayed in our case, aggressive invasive management of the airway is often demanded.[5,8]

Cervical spine immobilization is very important, especially in the light of trauma. The physician should assume pathology until proven otherwise. A systematic approach to detect a cervical pathology is strongly recommended, e.g. repeated clinical evaluation of the trauma patient according to advanced trauma life support algorithms.[9] Early diagnosis of the cervical fracture and early cervical spine immobilization might have prevented aggravation of the retropharyngeal hematoma. In this case, the lack of conscience during resuscitation, the subsequent surgical procedures, and the intensive care treatment underline the need for ongoing effective inline stabilization, ensured by a collar and later on, by a halo fixator. While the airway obstruction by the retropharyngeal hematoma was present before resuscitation and intubation attempts, the clinical course of our case was strongly suggesting that the retropharyngeal hematoma was caused by the initial trauma. Nevertheless, during the intubation of trauma patients, in-line stabilization is an universal precaution against additional hematoma formation and neurological damage to the spinal cord.

The unanticipated difficult airway situation was extremely challenging in this case. The utilization of all airway management steps with conventional laryngoscopic intubation, supraglottic airway, and fiberoptic device failed at least to maintain oxygenation and secure the patient’s airway. Ultimately, a “cannot intubate, cannot ventilate” situation developed. Therefore, a surgical airway was imperative. However, all participants had full panel airway management training in conventional orotracheal intubation, alternative airway devices, fiberoptic intubation, and emergency cricothyroidotomy. Unfortunately, the lack of anatomical landmarks made emergency cricothyroidotomy as the usual surgical airway not a promising approach.[10] Surgical tracheotomy was required. The immediate presence of a surgeon capable to master the difficult anatomical situation quickly shortened the time to a re-oxygenation. The patient’s outcome, regrettably, was characterized by the neurological impairment, most likely attributable to the hypoxic state. Nonetheless, in our case, the imminent risk for neurological damage would not have justified an aggressive approach to secure the patient’s airway.

Emergency medicine is no independent medical speciality in the country of the case’s origin, so that the fact that only anesthesiologists and surgical specialities were involved is no prejudice about the role of emergency physicians in trauma airway management in other countries. Data from the literature indicates that both anesthesiology and emergency medicine have comparable skills in the management of the trauma airway.[11] However, certainly, the case could be seen as an example for beneficial multidisciplinary airway management. Without the closed chain of survival and the presence of appropriate equipment, the patient’s life would not have been preserved. It reminds every physician working in emergency medicine to keep airway management skills up to date and prepare even for the rare and demanding situation of the surgical airway. Moreover, difficult airway equipments should be readily available. The spectrum of airway management devices is growing. However, local algorithms based on the provision of alternative laryngoscope blades, at least one supraglottic airway device, a flexible fiberoptic device, and material for the invasive, surgical airway should be implemented.[10]

## CONCLUSION

Difficult airway management for a retropharyngeal hematoma as described here must be quick and resolute. The immediate need for a surgical airway is highly likely in a case of upper airway obstruction. Therefore, preparedness for any difficult airway situations in emergency departments is indispensable. The 24-h presence of trained staff and available equipments for the unanticipated difficult airway in the emergency room proved to be a basic prerequisite, and should be integrated into a general hospital concept for a difficult airway. This should include preparations for the multidisciplinary action regarding the surgical airway and might be trained in advance.
